# Celluloid

**Published:** 1884-02

**Authors:** 


### CELLULOID.

Dentures made of this material when once broken are not easily mended. If a new piece be put in under heat and pressure, it will hold for a time, and then it separates. The only way to get approximate security in attaching a new piece, is to accurately fit it into a groove or dove-tail, soften the edges of both the plate and the new piece with a saturated camphor solution, and then apply pressure while the piece is cold, until a union between the parts is secured. After this the heat may be applied, and the piece pressed home.

But a better and surer way to repair a celluloid plate is to run plaster of paris into the palatal portion, and then cover the lingual and buccal surfaces with the thin sheet wax used in the manufacture of artificial flowers, after which it is invested in the flask, placed in the packing machine and heated until the old plate can be easily removed from the teeth. A new blank is then packed into this mould. The sheet wax referred to gives about the added thickness necessary for finishing. Of course more can be added in any place if needed.

